# *In Vitro* Corrosion and Cytocompatibility of ZK60 Magnesium Alloy Coated with Hydroxyapatite by a Simple Chemical Conversion Process for Orthopedic Applications

**DOI:** 10.3390/ijms141223614

**Published:** 2013-12-03

**Authors:** Bing Wang, Ping Huang, Caiwen Ou, Kaikai Li, Biao Yan, Wei Lu

**Affiliations:** 1 Affiliated Tongji Hospital, Tongji University, Shanghai 201804, China; E-Mail: 1020060044@tongji.edu.cn; 2 Shanghai Key Laboratory of D&A for Metal-Functional Materials, Tongji University, Shanghai 201804, China; E-Mails: 1233428@tongji.edu.cn (P.H.); 1020060059@tongji.edu.cn (K.L.); yan_biao@tongji.edu.cn (B.Y.); 3 Key Laboratory of Construction and Detection of Guangdong Province, Southern Medical University, No.1023, Shatai Nan Road, Guangzhou 510515, China; E-Mail: oucaiwennfy@gmail.com

**Keywords:** magnesium, hydroxyapatite, coating, corrosion, biocompatibility

## Abstract

Magnesium and its alloys—a new class of degradable metallic biomaterials—are being increasingly investigated as a promising alternative for medical implant and device applications due to their advantageous mechanical and biological properties. However, the high corrosion rate in physiological environments prevents the clinical application of Mg-based materials. Therefore, the objective of this study was to develop a hydroxyapatite (HA) coating on ZK60 magnesium alloy substrates to mediate the rapid degradation of Mg while improving its cytocompatibility for orthopedic applications. A simple chemical conversion process was applied to prepare HA coating on ZK60 magnesium alloy. Surface morphology, elemental compositions, and crystal structures were characterized using scanning electron microscopy, energy dispersive spectroscopy, and X-ray diffraction, respectively. The corrosion properties of samples were investigated by immersion test and electrochemical test. Murine fibroblast L-929 cells were harvested and cultured with coated and non-coated ZK60 samples to determine cytocompatibility. The degradation results suggested that the HA coatings decreased the degradation of ZK60 alloy. No significant deterioration in compression strength was observed for all the uncoated and coated samples after 2 and 4 weeks’ immersion in simulated body fluid (SBF). Cytotoxicity test indicated that the coatings, especially HA coating, improved cytocompatibility of ZK60 alloy for L929 cells.

## 1. Introduction

Metal materials such as titanium and stainless steel are widely used in implant applications. Potential defects with the use of these materials are the introduction of toxic ions to the organism [1–4], stress shielding effect [5], and the requirement to be removed with secondary surgery [6]. Among the solutions proposed to overcome these drawbacks, the adoption of biodegradable materials seems to be very promising. Polymers, such as polylactic acid (PLA) or polyglycolic acid (PGA), can be used to design implants because of their biodegradability. However, their low mechanical properties eclipse their advantages.

Recently, particular attention has been devoted to the use of magnesium alloys as a potential matrix material for designing implants [7–11]. Magnesium and its alloys offer intriguing solutions to existing issues with conventional metal implant materials, with approximate mechanical properties to that of human bone, degradability, and good biocompatibility of both the metal itself and the corrosion products [10,12–15]. Moreover, Mg ions can stimulate osteoblastic cell responses [16]. Therefore, magnesium alloy can enhance the osteoplastic activity around the degrading implants and finally lead to a complete replacement of the implant by bone tissue [11,17]. Unfortunately, although magnesium alloys have been investigated as implants for years, commercial implants are still unavailable [18]. The high degradation rate is the main limitation [17,19,20]. The corrosion resistance of magnesium alloy must be improved in order to widen the period of time when the scaffolding action of the implant is effectively exerted.

Strategies to overcome this drawback include changing the composition of the alloy and tailoring its microstructure, adopting surface modification (e.g., coatings) to temporarily insulating the bulk metallic structure or changing the implant design. Among all the solutions proposed as yet, calcium phosphate coating is the most effective way to improve the corrosion resistance and potentially improve biological activity of magnesium-based implants [21]. As the end product of the biological mineralization process, hydroxyapatite (HA) is currently used as a biomedical material in the form of coating or powder and exhibits excellent biocompatibility and bioactivity due to its chemical and structural similarities to human bone. Furthermore, HA has been reported to have the lowest solubility among all the calcium phosphate phases in physiological environments [22]. Therefore, preparing HA coating on magnesium alloy can potentially impede the corrosion of substrate in body fluid. Until now, HA coating on Ti-based implants has been deeply studied over the years [23–25]. However, research on preparation of HA coating on magnesium alloy has just begun. There are various techniques for depositing Ca-P coating onto Mg substrates: biomimetic deposition, electrodeposition, hydrothermal treatment and alkali-heat treatment [26–29]. Though many methods for fabricating HA coatings are available, the chemical conversion is a more convenient and simple way to prepare Ca-P coating on magnesium alloy, and can be conducted at room temperature. Several literatures report the fabrication of HA-based coating on Mg-based substrates via chemical conversion and subsequent alkaline treatment [29,30]. The alkaline treatment will lead to not only the formation of HA phase but also the corrosion of Mg substrate.

Thus, the present work attempts to prepare Ca-P coating on ZK60 alloy using a simple chemical conversion process. Instead of alkaline treatment, simple heat treatment was used to transform the as-deposited Ca-P coating to HA coating. The microstructure and composition as well as the *in vitro* corrosion behavior and cytocompatibility of the samples were investigated. The HA coated ZK60 alloy was demonstrated to be having good corrosion resistance and cytocompatibility.

## 2. Results and Discussion

### 2.1. Structures and Morphologies of the Coating

**Figure 1.**
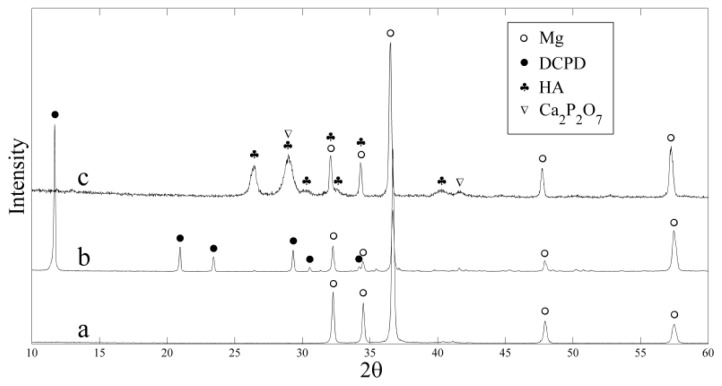
XRD patterns of the substrate and the coatings: (**a**) uncoated ZK60 alloy; (**b**) as-deposited coating; and (**c**) heat-treated coating.

Figure 1 illustrates the XRD patterns of the magnesium alloy substrate, as-deposited coating and heat-treated coating. Taking no account of the substrate peaks, all the other peaks in pattern (b) can be attributed to the Brushite (dicalcium phosphate dehydrate, DCPD; CaHPO_4_·2H_2_O). The peak at 11.68° 2θ represents the typical reflection of (020) plane of DCPD crystal. The heat-treated coating mainly consists of hydroxyapatite (HA), as shown in pattern (c). Furthermore, the peak at 26° 2θ represents the reflection of (002) plane of HA. DCPD is precursor of HA and can be transformed into HA by alkaline treatment [31]. Here, we proved another way to obtain HA coating by using direct heat-treatment process. In addition, Ca_2_P_2_O_7_ was also found in the pattern (c), which was formed during the heat treatment process. Furthermore, it can be seen that the peaks of the heat-treated coating are broadened, indicating the existence of some structural disorder or amorphous phases in the coating.

**Figure 2.**
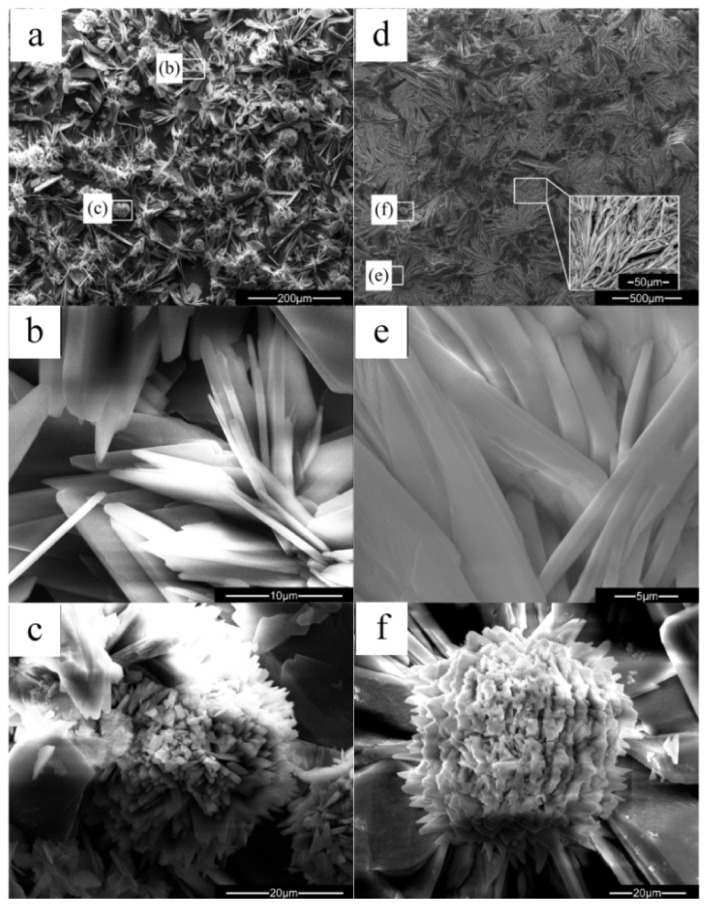
SEM morphologies of the dicalcium phosphate dehydrate (DCPD) coating (**a**–**c**) and the hydroxyapatite (HA) coating (**d**–**f**).

**Figure 3.**
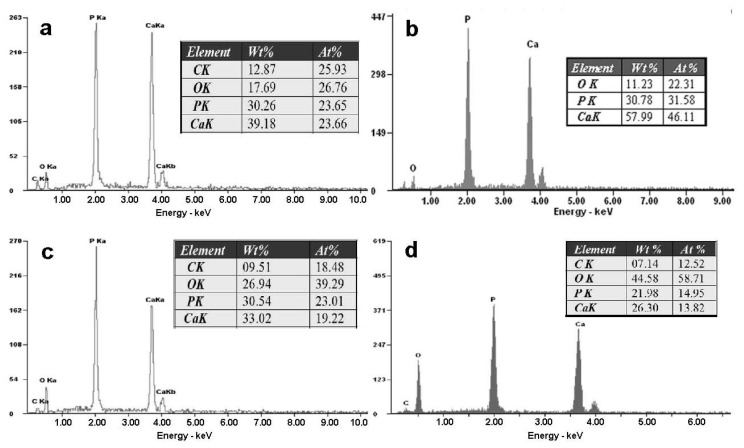
Energy dispersive spectroscopy (EDS) for the different morphologies in the DCPD coating and the HA coating: (**a**) and (**b**) correspond to Figure 2b and Figure 2c, respectively; (**c**) and (**d**) correspond to Figure 2e and Figure 2f, respectively.

Figure 2 presents the SEM morphologies of the DCPD coating (Figure 2a–c) and HA coating (Figure 2d–f) formed on the ZK60 substrate. As is shown in Figure 2a, the DCPD coating shows two kinds of morphologies. Most of the coating display flake-like morphology with fine crystal size, which is illustrated in Figure 2b. This morphology is very similar to the DCPD coating prepared by electrodeposition in Song’s study [32], although the chemical deposition process applied in this research is much simpler than the electrodeposition. The EDS in Figure 3a shows that the Ca/P molar ratio of the flake-like structure is 1.0. The others appear chrysanthemum-like morphology with radiated columnar structure, as shown in Figure 2c. The Ca/P molar ratio is a little lower than that of DCPD. The HA coating showed two major morphologies: large plate-like structure (Figure 2e) and fine dendritic structure (marked in Figure 2d), which is also similar to the HA coating prepared by electrodeposition [33]. EDS in Figure 3c showed the Ca/P molar ratio is about 1.46, indicated that the coating was Ca-deficient hydroxyapatite. Chrysanthemum-like structure (Figure 2f) was also observed in the heat-treated coating, and the Ca/P molar ratio was ~1.0. It is possible that the chrysanthemum-like structure is Ca_2_P_2_O_7_. C element was detected in both the coatings possibly caused by the dissolution of CO_2_ in the air. CO_3_^2−^ might substitute the PO_4_^3−^ in the apatite, producing a coating that has a similar composition with the biological apatite from natural bone mineral and was beneficial to the biocompatibility [34].

The DCPD coating is not dense enough and many pores can be found among the crystals. Fewer pores are found in HA coating and may provide better protection from corrosion. On the other hand, these pores are likely to be beneficial for the infiltration of new bone tissues into the implants and accelerating the healing of the damaged bones [35,36]. All the flake-like, plate-like and dendritic structures of the coatings were helpful for bone growth, since the inorganic apatite in bone has a plate-shaped morphology [37]. In addition, after heat treatment, the crystal size is enlarged which may be due to phase transition and recrystallization. Moreover, no visible cracks due to thermal or other stress could be found in the coatings.

### 2.2. Corrosion Behavior in SBF

#### 2.2.1. pH-Value Changes of the SBF Solution

**Figure 4.**
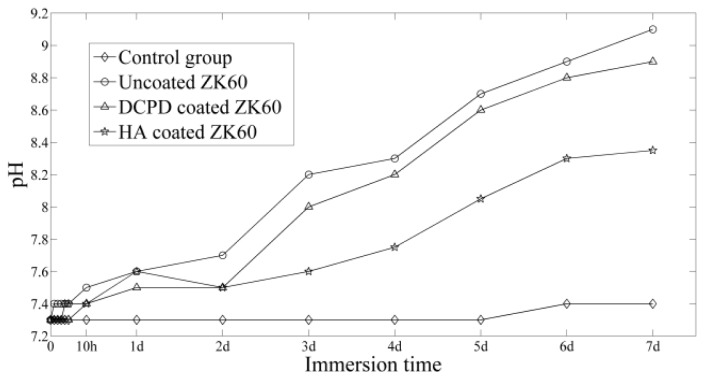
pH changes of the SBF during 7 day’s immersion tests.

*In vitro* immersion tests were conducted to evaluate the degradation behavior of the samples. Figure 4 shows the variation of the pH value of SBF as a function of immersion time. In general, there is a pH increase for all the groups with samples. However, the pH values of the solutions corresponding to the coated ZK60 increased at a smaller rate compared with the uncoated sample. As is known that the increase of the pH values in SBF were mainly resulted from the corrosion of Mg or its alloys [38], it can be concluded that corrosion rate of coated alloys is lower than that of uncoated sample. Furthermore, HA coated ZK60 possessed the lowest pH increase among the three groups, suggesting that HA coating provided the best protection for the substrate. A physiological condition with high pH values is not compatible for cell growth [39]. The manipulation of pH values is a critical problem for using Mg alloys as orthopedic biomaterials [38]. Consequently, HA coated ZK60 can cater for this requirement with the best corrosion resistance and biocompatibility.

#### 2.2.2. Corrosion Morphologies and Products of the Samples

**Figure 5.**
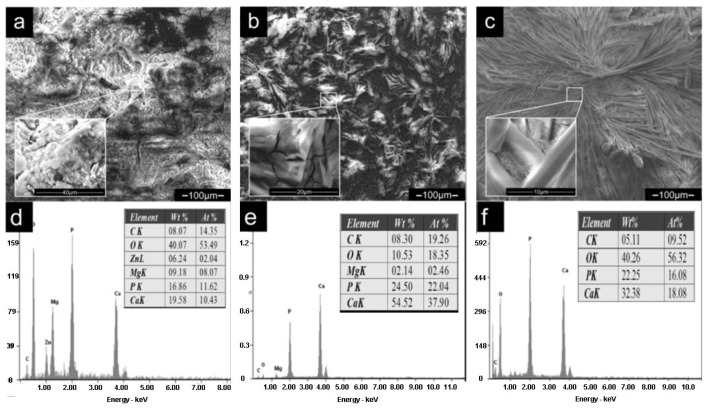
Corrosion morphologies and corresponding EDS of corrosion products: (**a**) and (**d**) for uncoated ZK60; (**b**) and (**e**) for DCPD coated ZK60; (**c**) and (**f**) for HA coated ZK60.

Figure 5 displays the surface morphologies and corresponding EDS of the samples after immersion tests. Both the uncoated and DCPD coated ZK60 present network-like cracked appearances due to corrosion. The uncoated ZK60 (Figure 5a) corroded seriously and some white precipitates were formed on the surface. The precipitates were mainly composed of Ca, P, O, C, Mg and Zn elements, suggesting that there was bone-like apatite formed on the surface. Similar results were obtained in Huan’s study [40].

After immersion, the morphology and composition of DCPD coating (Figure 5b,e) changed significantly. The flake-like morphology disappeared and the Ca/P ratio increased from 1.0 to 1.7. This indicated that DCPD coating was transformed into HA. Two factors might have contributed to this phenomenon: (1) DCPD is considered as a precursor for HA precipitation and a further pH increase during the immersion can promote HA nucleation [41]; (2) The dissolution of DCPD leads to the release of calcium and phosphate ions and these ions in turn re-precipitated in the form of HA [42]. However, no HA formed on the surface of the DCPD coated Mg alloy after immersion in SBF for one week in similar researches [32,43]. Different results are obtained in different studies [32,41–43]. Further study is still desirable to evaluate the degradation behavior of DCPD coating.

**Figure 6.**
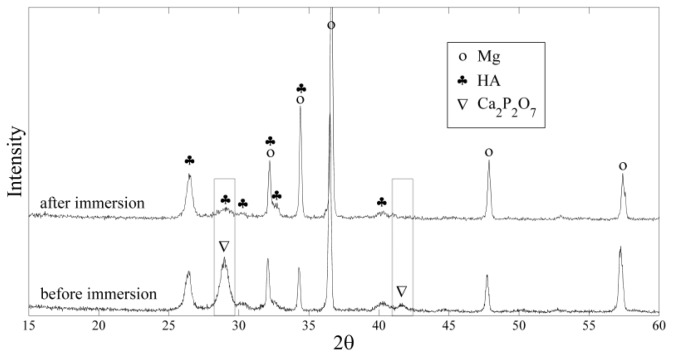
Comparison between the XRD patterns of HA coating before and after immersion test.

Figure 5c,f shows the corrosion morphology and composition of HA coating after seven days’ immersion. Compared with the HA coating before immersion, no obvious changes in the morphology were observed. The coating still covered the ZK60 substrate completely and could provide protection for the substrate from corrosion. In the boundary between the HA grains and on the surface of some grains, villous structure was developed. This structure will block up the boundary and consequently improve the coating’s corrosion resistance. No visible cracks and evident corrosion phenomenon can be seen on the HA coated sample after immersion testing, in comparison with uncoated and DCPD coated alloy, suggesting that HA coating had the best corrosion resistance. XRD was also used to examine the microstructure changes for HA coating after immersion test. As shown in Figure 6, the coating after immersion still composed of HA. However, compared with the coating before immersion, the peaks of Ca_2_P_2_O_7_ disappeared, which may be due to the high solubility of Ca_2_P_2_O_7_ in SBF.

#### 2.2.3. Electrochemical Behavior in SBF

**Figure 7.**
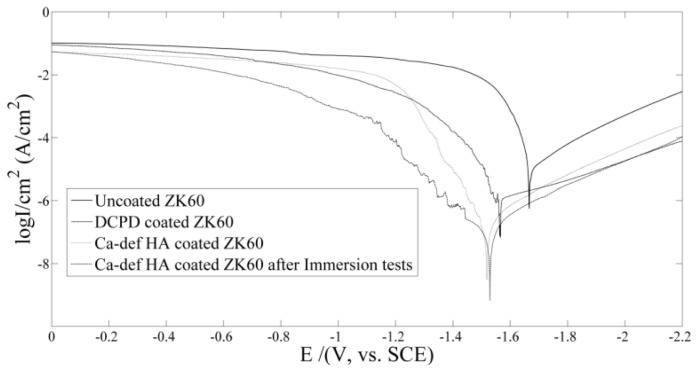
Potentiodynamic polarization curves of the samples obtained in SBF at 37 °C.

The potentiodynamic polarization curves of the samples obtained in SBF are shown in Figure 7 and the corresponding electrochemical data are given in Table 1. Compared with uncoated ZK60, the *E*_corr_ values of the DCPD coated ZK60 and HA coated ZK60 shift towards noble direction by 100 and 148 mV, respectively. Meanwhile, the corrosion current density *i*_corr_ was also decreased with the addition of the coatings, with 1 order for the DCPD coating and 2.5 orders for the HA coating. The result indicated that the corrosion resistance of the ZK60 alloy is improved by the coatings and the HA coating is more protective than DCPD. Given that the HA coating kept its integrity after immersion testing, its electrochemical properties after immersion was also tested. Results show that both the *E*_corr_ and *i*_corr_ of HA coated ZK60 stayed in the same level before and after the seven day immersion test. At the same time, the polarization was slightly enhanced after immersion, indicating slight improvement in corrosion resistance of HA coating. This might be due to the villous formations between the HA grains observed in Figure 5c.

**Figure 8.**
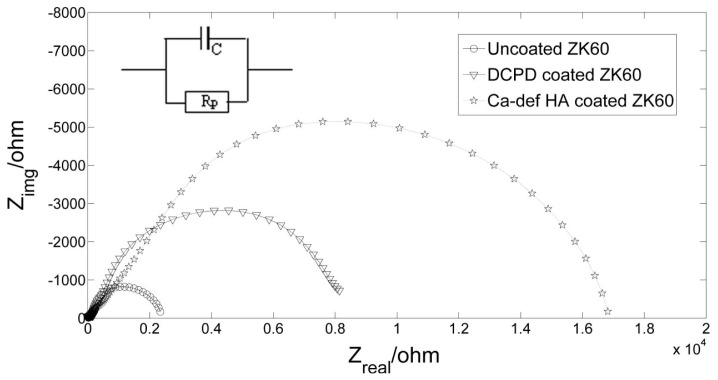
Nyquist curves of the samples obtained in SBF at 37 °C.

The EIS data of the uncoated ZK60 and coated ZK60 are presented in Nyquist curves in Figure 8. All curves show only one capacitance loop. Literature suggested that the diameter of the capacitive loop was directly proportional to the surface film resistance (*R*_f_) for mass transfer and charge transfer resistance (*R*_CT_), and the sum of *R*_f_ and *R*_CT_ was taken as polarization resistance (*R*_p_) [45]. The HA coated sample shows the largest diameter as shown in Figure 8, suggesting that HA coating had the maximum *R*_p_ value. The larger the *R*_p_ is, the better the corrosion resistance of the coating is. The estimated *R*_p_ values obtained from the Nyquist curves are shown in Table 1. The polarization resistance of the DCPD coated ZK60 is four times as high as uncoated ZK60, meanwhile, eight times for the HA coated ZK60.

The improvement in corrosion resistance will greatly reduce the initial biodegradation rate of the implants, which is essential for maintaining the implant’s mechanical strength during the bone reunion period. Furthermore, Song [46] suggested that a potential magnesium-based implant should possess a six times’ lower hydrogen evolution rate than AZ91 alloy. Comparing present electrochemical data of the coated ZK60 alloy with that of AZ91 alloy, the improvement in the corrosion resistance is very encouraging, especially for the HA coated ZK60.

### 2.3. Biocompatibility

**Figure 9.**
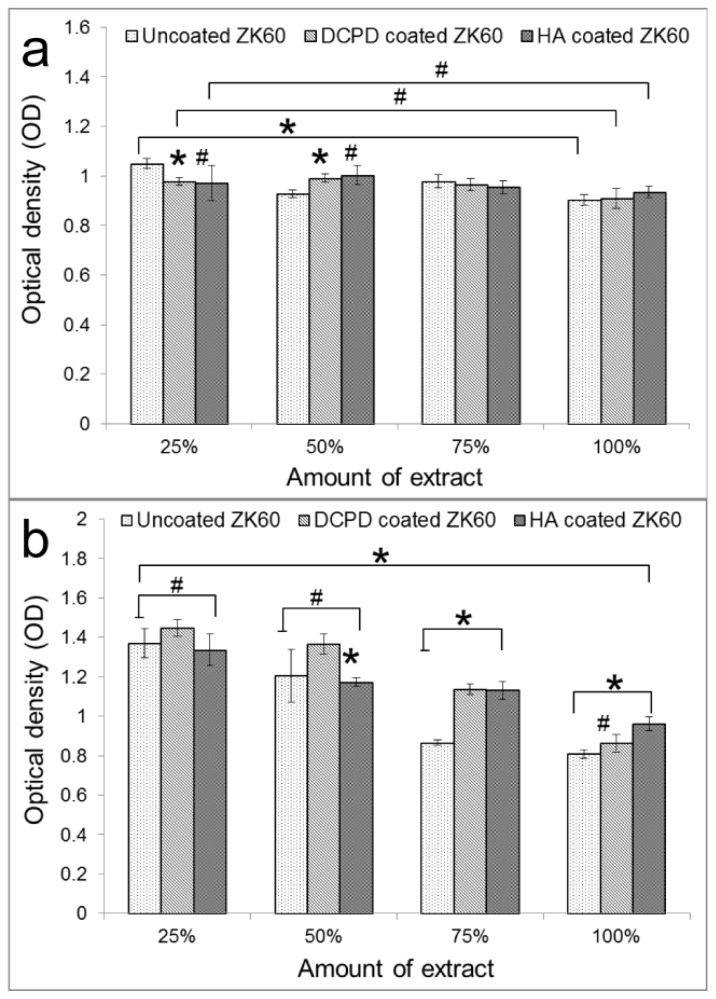
L-929 cell viability expressed by the optical density of the cells using the MTT assay after (**a**) one day and (**b**) three day incubation in uncoated ZK60, DCPD coated ZK60 and HA coated ZK60 extraction media. (*p < 0.05, ^#^*p* > 0.05).

Figure 9 shows the L-929 cell viability cultured in the individual extraction mediums of uncoated, DCPD coated and HA coated ZK60 alloy for one and three days. After one day of culture (Figure 9a), no significant differences were observed for both the DCPD coated and HA coated ZK60 at extract concentrations of 25%, 50%, 75% and 100% (ANOVA on ranks, *p* > 0.05). However, ANOVA testing showed that there was significant difference between 25% extract and 100% extract for uncoated ZK60 (*p* < 0.05). The amount of extract of the uncoated ZK60 showed clearer influence on cell viability than the coated ZK60 after one day of incubation. The uncoated and coated ZK60 alloy showed similar cell viability at higher concentration (75% and 100%).

After three days of culture (Figure 9b), a decrease of absorption could be observed for all materials with an increase in the amount of extract. ANOVA tests indicated there were significant differences for all the samples between 25% and 100% (*p* < 0.05), while no significant differences were observed for HA coated ZK60 at 25%–75% (*p* > 0.05). The amount of extract influenced the cell viability for all the samples. However, for the HA coated ZK60, the influence was not so considerable to significantly decrease the cytocompatibility of the materials. In addition, there were no significant differences for the three groups at 25% and 50% extract (*p* > 0.05). However, the absorption values for uncoated ZK60 were significantly lower than the coated samples at 75% and 100% (*p* < 0.05), suggesting that the cytocompatibility of the substrate was improved by the coatings, especially by the HA coating.

From the analysis of the above results, it can be concluded that, with an increase of incubation time and the amount of extract, HA coating shows the best cytocompatibility. Studies have proved that Mg-Zn-Zr alloy possessed a good cytocompatibility [40]. In addition, according to ISO 10993–5: 1999, the cytotoxicity of the uncoated ZK60 in this study is also at a level of biosafety suitable for cellular applications. The coatings prepared in this study can further improve the cytocompatibility of ZK60 alloy, which demonstrates its qualification as biodegradable materials.

Previous *in vitro* cytotoxicity studies on magnesium alloys have suggested that a lower corrosion resistance leads to a higher pH value caused by corrosion, which finally reduces cell viability [40]. It is expect that, with enhanced corrosion resistance by the coating, the coated ZK60 will have higher cytocompatibility than the uncoated one. This expectation was demonstrated by the MTT results. In immersion tests, the pH values increased not so fast in the first day, leading to similar viabilities among the three groups. After three days of immersion, the increase of pH values was differentiated with the following sequence: uncoated ZK60 > DCPD coated ZK60 > HA coated ZK60. This resulted in an exactly opposite trend in MTT results, demonstrating the improvement of corrosion resistance and cytocompatibility of ZK60 by the coatings.

## 3. Experimental

### 3.1. Preparation of HA Coating

The magnesium alloy used in the experiment was casted Mg-Zn-Zr alloy (ZK60), with the major alloying elements of approximately 5.5% Zn and 0.5% Zr. ZK60 was cut into rectangular samples with dimensions of ~10 × 10 × 5 mm^3^. Each sample was mechanically ground up to a 2000 grid, cleaned using deionized water and ethanol, and then dried in open air. For the preparation of HA coating, the samples were immersed in a beaker containing 0.05 M Ca(NO_3_)_2_·4H_2_O and 0.03 M NaH_2_PO_4_·2H_2_O for 48 h at room temperatures. Then, the samples were removed from the solution and heated at 350 °C for 3 h. Finally, the treated samples were rinsed using deionized water and subsequently dried at room temperatures. The uncoated alloys and the samples before heat treatment were also tested.

### 3.2. Morphology and Structure Characterization

The morphology of the coating was observed using Field Emission Scanning Electron Microscopy (FE-SEM, Quanta 200, FEI Company, OR, USA). The chemical composition of the coating was determined using energy dispersive spectroscopy (EDS, Quanta 200, FEI Company). The phase structure was investigated using X-ray diffraction (XRD, D/max2550, Rigaku, Japan) with a Cu K_α_ target operated at 40 keV and 40 mA, and patterned for raising angles 10° < 2*θ* < 60° with a step size of 0.02°.

### 3.3. *In Vitro* Corrosion Tests

In order to evaluate the degradation properties of the HA coated magnesium alloy, *in vitro* immersion tests and electrochemical measurements were conducted in a simulated body fluid (SBF) [28,29], which was composed of NaCl 8.0 g·L^−1^, CaCl_2_ 0.14 g·L^−1^, KCl 0.4 g·L^−1^, NaHCO_3_ 0.35 g·L^−1^, Glucose 1.0 g·L^−1^, MgCl_2_·6H_2_O 0.1 g·L^−1^, Na_2_HPO_4_·2H_2_O 0.06 g·L^−1^, KH_2_PO_4_ 0.06 g·L^−1^, MgSO_4_·7H_2_O 0.06 g·L^−1^, pH 7.4.

#### 3.3.1. Immersion Tests

Immersion tests were carried out in SBF with the temperature kept at 37 ± 0.5 °C using a water bath. Each of the coated and uncoated samples was individually immersed into 100 mL SBF solution for 7 days. The pH values of the solution were recorded during the immersion process at different times, with a blank SBF solution as control group. Changes in the surface morphologies of the samples after immersion were observed using SEM, while the compositions were investigated using EDS.

#### 3.3.2. Electrochemical Measurements

Electrochemical measurements were carried out using a standard three-electrode cell, with saturated calomel as the reference, a platinum electrode as the counter and the sample as the working electrode. The temperature was kept at 37 ± 0.5 °C and the dimension of the working electrode was 1 cm^2^. The EIS experiments were performed at the open circuit potential with AC amplitude of 10 mV over a frequency range from 10^5^ to 10^−2^ Hz. Potentiodynamic polarization tests (CHI600, Shanghai, China) were obtained at a scan rate of 5 mV/s. Before the beginning of the potentiodynamic polarization and EIS tests, the samples were soaked in the solution for 20 min to establish the free corrosion potential.

### 3.4. Cytotoxicity Assessments

#### 3.4.1. Cell Culture

Murine fibroblast L-929 cells were purchased from cell bank of Chinese Academy of Sciences, Shanghai (Shanghai, China). The cells were cultured in Dulbecco’s modified Eagle’s medium (DMEM, high glucose) (Gibco, Shanghai, China), supplemented with 10% fetal bovine serum (FBS) in a humidified incubator at 95% relative humidity and 5% CO_2_ at 37 °C. Cells were passaged at about 80%–90% confluency. For the experiments, cells after the fifth passage were used.

#### 3.4.2. Preparation of Extracts of Samples

Cytotoxicity was determined by indirect contact. Extracts were prepared according to ISO 10993-12:2004. Three days prior to the cell seeding, the specimens were incubated in cell culture medium for 72 h under cell culture conditions in an oscillation incubator. The relation between the sample weight and extract medium was 1.25 cm^2^/mL. The obtained extracts were applied to the cells in four different concentrations (100%, 75%, 50% and 25%). The dilution of the extracts was made with pure cell culture medium.

#### 3.4.3. MTT Tests on Extracts

The cytotoxicity assays was performed in 96-well plates. L-929 cells were seeded with a cell density of 1500 cells/well and incubated for 24 h to allow cell attachment. Then, the medium was replaced by 100 μL extraction medium. After incubation in a humidified atmosphere for 1 and 3 days, 20 μL 3-(4,5-dimethylthiazol-2-yl)-2,5-diphenyltetrazolium bromide (MTT) (Sigma, Shanghai, China) with a concentration of 5 mg·mL^−1^, was added into each well. Then, the 96-well plate was incubated for 4 h under cell culture conditions. Subsequently, 150 μL dimethyl sulphoxide (Sigma, Shanghai, China) was added to the cells by replacement of the extracts, to dissolve the formazan crystals. Optical density (OD) measurements were conducted at 570 nm using a PowerWave HT Microplate Spectrophotometer (BioTek, Shanghai, China).

## 4. Conclusions

DCPD and HA coating was successfully prepared on ZK60 alloy by a simple chemical conversion process and heat treatment. In comparison to the non-coated ZK60 sample, both the coated samples showed a significantly decreased degradation rate, indicating that the Ca-P coatings protected the ZK60 substrate from rapid degradation. Moreover, the HA coating provided a better protection than the DCPD coating, which was indicated in the immersion test and electrochemical test. Cytotoxicity test suggested that the HA coating improved the cytocompatibility of ZK60 alloy for L929 cells. These findings indicate that HA-coatings prepared in the current study are promising for controlling the biodegradation and improving the cytocompatibility of ZK60 Mg-based orthopedic implants and devices. However, further cell and animal studies are needed in order to translate HA-coated ZK60 implants to clinical applications.

## Figures and Tables

**Table 1.** Electrochemical data extracted from the polarization curves and Nyquist curves.

Sample	*E*_corr_ (mV)	*i*_corr_ (μA/cm^2^)	*R*_p_ (ohms)
Uncoated ZK60	−1666	35.39	2361
DCPD coated ZK60	−1566	3.564	8314.4
HA coated ZK60	−1518	0.1408	16949
HA coated ZK60 after immersion test	−1529	0.107	–
AZ91 *	−1713	65.7	–

* The electrochemical data for AZ91 alloy are taken from the literature reported by Kannan and Raman [44].
